# DEVELOPING A TYPOLOGY OF SENIOR CENTERS: CONCEPTUAL AND METHODOLOGICAL CONSIDERATIONS

**DOI:** 10.1093/geroni/igae098.3120

**Published:** 2024-12-31

**Authors:** Ceara Somerville, Jan Mutchler

**Affiliations:** University of Massachusetts Boston, Boston, Massachusetts, United States; University of Massachusetts Boston, Boston, Massachusetts, United States

## Abstract

Senior centers are important resources for older adults and their families, intended to serve their communities by tailoring programs and services to meet the needs and interests of those in their local environment. The network of senior centers across the nation and within states is heterogenous, but there is limited research focused on senior centers’ organizational capacity. This project uses a novel dataset from a comprehensive database of all senior centers in Massachusetts (n=342) to explore the commonalities and differences in models of senior center operations. A factor-cluster approach was applied to a subsample of 278 senior centers. Nearly 40 indicators of organizational goals, structure, people, resources, and policies were included in a nonlinear principal components analysis, resulting in 11 standardized scales representing components of organizational capacity (e.g., leadership, governance, core programs and services). These scores were then entered into a two-step cluster algorithm to determine the optimal grouping of senior centers into distinct types of operational models. Three clusters emerged; the most notable differences between them occurred on measures of wellness programs, core programs and services, staff capacity, space capacity, and social and financial resources. Differences between clusters on several demographic characteristics of the community (e.g., population size and density, racial and ethnic composition) were observed, providing empirical support of senior centers operating in response to their local populations. Senior centers are locally focused, but identifying common operational types and their relation to the wider community can inform research, policy, and practice to better support the network.

